# Cerebral Venous Outflow Implications in Idiopathic Intracranial Hypertension—From Physiopathology to Treatment

**DOI:** 10.3390/life12060854

**Published:** 2022-06-08

**Authors:** Sorin Tuță

**Affiliations:** 1 Department of Neurology, “Carol Davila” University of Medicine and Pharmacy, 050471 Bucharest, Romania; sorin.tuta@umfcd.ro; 2 Department of Neurology, National Institute of Neurology and Neurovascular Diseases, 041914 Bucharest, Romania

**Keywords:** idiopathic intracranial hypertension, pseudotumor cerebri, cerebral venous sinus stenosis, magnetic resonance venography, cerebral venous sinus stenting, optical nerve sheath fenestration

## Abstract

In this review, we provide an update on the pathogenesis, diagnosis, and management of adults with idiopathic intracranial hypertension (IIH) and implications of the cerebral venous system, highlighting the progress made during the past decade with regard to mechanisms of the venous outflow pathway and its connection with the cerebral glymphatic and lymphatic network in genesis of IIH. Early diagnosis and treatment are crucial for favorable visual outcomes and to avoid vision loss, but there is also a risk of overdiagnosis and misdiagnosis in many patients with IIH. We also present details about treatment of intracranial hypertension, which is possible in most cases with a combination of weight loss and drug treatments, but also in selected cases with surgical interventions such as optic nerve sheath fenestration, cerebral spinal fluid (CSF) diversion, or dural venous sinus stenting for some patients with cerebral venous sinus stenosis, after careful analysis of mechanisms of intracranial hypertension, patient clinical profile, and method risks.

## 1. Introduction

Idiopathic intracranial hypertension (IIH) is considered a relatively rare disorder characterized by elevated intracranial pressure (ICP) without a clear cause, remaining a diagnosis of exclusion, after other known situations associated with raised ICP have been evaluated. Several approaches have proposed different names of this entity according to the knowledge and understanding at that point in time. In 1904, ‘pseudotumor cerebri’ was proposed by Nonne, and in 1937 Walter Dandy [1] described it under the name ‘intracranial pressure without brain tumor’; later, in the 1950s, it was named benign intracranial hypertension. Unfortunately, if left untreated, the disorder can lead to prolonged headache, pulsatile tinnitus, reactive depression with reduced quality of life, but also substantial visual morbidity, including complete blindness. All these reasons changed the opinion on benign intracranial hypertension, and the actual term of IIH is the most used. Pseudotumor cerebri is still used for the secondary forms of intracranial hypertension, after cerebral magnetic resonance imaging (MRI) excludes an intracranial mass lesion. Exclusion of a secondary cause of intracranial hypertension is part of the IIH diagnosis workflow, and from this point of view the cerebral venous and sinus thrombosis is a much better known and accepted cause of intracranial hypertension: 10% of patients with cerebral venous thrombosis developed chronic intracranial hypertension during follow-up in one study [2] and dural sinus thrombosis was identified in 26% of patients with initial IIH, but in the last two decades, more evidence has emerged toward a similar role of dural venous sinus stenosis without thrombosis [3].

Although early diagnosis and treatment are crucial for favorable visual outcomes in IIH patients, there is also a risk of overdiagnosis and misdiagnosis in as many as 40% of patients initially diagnosed with IIH [4]. Patients with a secondary cause of intracranial hypertension (with the exception of those with a cerebral mass lesion) belong to the category of pseudotumor cerebri, but on the other hand, in 355 patients analyzed with CT angiography in a recent study [5], the prevalence of unilateral transverse sinus stenosis or hypoplasia in the general population was up to 33%, 5% for bilateral transverse sinus stenosis, and 1% for unilateral stenosis with contralateral hypoplasia. Therefore, a diagnosis of cerebral sinus stenosis is important, but to prove there is a causal link with intracranial hypertension is sometimes challenging and requires measurement of the pressure gradient at the level of the stenosis with all other plausible causes to be excluded. This is the reason why in the present review the venous sinus stenosis is often discussed in relation to idiopathic intracranial hypertension and not considered a priori to belong to the pseudotumor cerebri category.

The process of collection and selection of published papers for this review started with a search of the PubMed and Cochrane Libraries for all peer-reviewed articles from 2002 to date with a combination of key words, including “idiopathic intracranial hypertension,” “pseudotumor cerebri”, and “benign intracranial hypertension”, and after that a second search was started for more narrow and detailed information about “cerebral glymphatic ”, ”cerebral lymphatic”, ”cerebral venous sinus stenosis”, ”cerebral venous sinus stenosis stenting”, “lumboperitoneal shunts and intracranial hypertension”, and “optical nerve sheath fenestration”. Other references from the articles that were identified in the initial search and important data for clinical studies of IIH treatment were also selected and reviewed for extraction of additional information or points of view.

In this synthetic review of the literature, we provide the latest information and points of view with respect to the pathogenesis, diagnosis, and management of adult idiopathic intracranial hypertension, and we highlight the progress made during the past decade regarding the mechanisms of venous outflow pathway obstruction, emphasizing the role of cerebral venous sinus stenosis, but also of its connection with the cerebral glymphatic and lymphatic network in genesis of IIH. We present details about treatment of intracranial hypertension related to venous sinus stenosis and IIH, which is possible in most cases using a combination of weight loss and drug treatments, but also surgical interventions such as optic nerve sheath fenestration, cerebral spinal fluid (CSF) diversion, and dural venous sinus stenting in selected cases of cerebral sinus stenosis, after careful analysis of mechanisms of intracranial hypertension, patient clinical profile, and risks.

## 2. Physiopathology of Idiopathic Intracranial Hypertension—A Connection between CSF, Cerebral Glymphatic, Lymphatic, and Venous Drainage

Starting with the historical hypothesis of Monro and Kelly, later completed by Magendie and Burrows, the skull was described as a rigid structure containing incompressible brain, and it was stated that the sum of the volume of brain, blood, and cerebrospinal fluid (CSF) is constant: an increase in one causes a decrease in one or both of the remaining two, but once the period of compliance due to the displacement of CSF or blood runs out, there is an exponential curve of a rise in intracranial pressure. An increased volume of the brain parenchyma is classically associated with cerebral edema (such as in large ischemic strokes, encephalitis, traumatic brain injury, and malignant tumors), or intraparenchymal expanding lesions such as hematomas or tumors, but this is not the main objective of this review [6,7].

The epithelial cells of the choroid plexus are the main source of CSF secretion, with a rate of 0.3–0.4 mL/min, while a secondary source is the interstitial fluid of the brain resulting from filtration through the blood–brain barrier, which is estimated to be of a much lower importance, but the overall volume of CSF is about 150–160 mL [6]. The difference in hydrostatic pressure between choroid plexus capillaries, epithelial cells, and ventricles also depends on blood pressure and represents an important factor for the net filtration process resulting in CSF production [7].

Both osmotic and hydrostatic pressure gradients contribute to CSF formation. As alterations to osmolarity modify water flux across the choroid plexus, the transport of ions across the blood–CSF barrier is important in the secretion process. In this process, ions are transported from circulating blood into the CSF via their respective transporters in the choroid cells’ walls, while water is likely transported by a combination of a transcellular process against an osmotic gradient by cotransporters and via tight junctions [6].

Although osmotic and hydrostatic pressure gradients between choroid plexus cells and ventricles could play a role for CSF secretion in some instances, this role is minor, and some cotransporter proteins may have a significant role. While aquaporin-1 (AQP1)-dependent water channels have an important role at the apical membrane of choroid cells, the water permeability at the basolateral membrane is dependent on other proteins, such as the glucose carrier GLUT1. At the same time, paracellular water transport through the tight junctions toward lateral spaces between epithelial cells is determined by proteins from the claudin family (most often claudin-1, -2, and -3, but also -9, -19, and -20) [7,8].

Water enters from interstitial space and the capillary pole into the choroid cells through the basolateral membrane and participates in CSF formation at the apical membrane of choroid plexus, with both processes mediated by aquaporin-1, and it can be upregulated by retinoids and glucocorticoids, which explains the implication of some medications in IIH [6,8].

The cotransporter proteins act at the basolateral membrane of epithelial cells (interstitial space border) and the apical membrane (CSF border of the epithelial cells), facilitating the movement of some ions with importance in CSF production, the Na^+^ gradient driving HCO_3_^−^ and Cl^−^ into the epithelial cells. The Na^+^–K^+^-ATPase pump and Na^+^–K^+^–2Cl^−^ cotransporter (NKCC1) are very important to these processes, but also the Na^+^–HCO_3_ cotransporter, with accumulation of HCO_3_^−^ and H^+^ from carbonic anhydrase activity. This derives the role of acetazolamide inhibition of carbonic anhydrase and the further reduction of CSF production by almost 50%, as well as the same effect of the NKCC1 inhibitor bumetanide [6,8,9,10].

The secretion and resorption of CSF must be balanced to maintain a constant volume of CSF of around 150–160 mL, whereby an excess amount will produce a rise in intracranial pressure through an increase of the total fluid content of the brain. There are several possibilities for CSF’s contribution to raised intracranial pressure. First, it could be an increased production with insufficient outflow, such as in tumors of the choroid plexus, where sometimes hydrocephalus can occur, whereas in idiopathic intracranial hypertension the ventricles remain a normal size. Another mechanism is the case of obstructive hydrocephalus that could result secondary to an obstruction along the CSF pathway, in contrast with non-obstructive hydrocephalus, where a decreased resorption is the cause of accumulation of CSF. Classically, the arachnoid granulations have the main role in CSF clearance. Absorption of CSF depends on the pressure gradient between the venous sinus and the subarachnoid space, so a rise in venous pressure needs a concomitant increase in CSF pressure to maintain absorption rates. There are instances such as subarachnoid hemorrhage or meningitis where a blockage of the arachnoid granulation and lymphatics by blood cells or fibrosis is produced, where reduced absorption of CSF could result in intracranial hypertension and communicating hydrocephalus, but in IIH such development is not visible [10,11].

Through a similar mechanism, both CSF hypercellularity, as seen in malignant meningitis, and high CSF protein in Guillian–Barré syndrome, can elevate ICP. Vitamin A deficiency can also lead to elevated ICP, with evidence of thickening of the extracellular matrix in the arachnoid villi [10,11].

Interstitial fluid of the brain is produced by fluid secretion and filtration at the blood–brain barrier level, being distributed between neurons, glial cells, and capillary cells within the parenchyma, while CSF fills the ventricles and subarachnoid space. There is an interaction between interstitial fluid and cerebrospinal fluid that takes place in the perivascular spaces surrounding the small penetrating vessels in the brain parenchyma, where a slow convective flow takes place through the glymphatic system [10,11].

From the subarachnoid spaces, the CSF enters the periarterial spaces, traveling from the cortex toward the deep white matter along the courses of the pial and the perforator arteries in a centripetal distribution [12]. This process of slow flow is passively driven by pressure gradients, including the difference in pressure during respiration, but actively by the arterial pulsations pump. It looks as though this process is more active during nighttime sleep. Another fraction of interstitial fluid derives from trans ependymal passing of CSF from cerebral ventricles and from the periventricular area, reaching periarterial spaces in a centrifugal fashion, a process mediated by aquaporin-4 (AQP4), expressed in the astrocytic end-feet from the structure of the blood–brain barrier. AQP4, which occupies ~50% of the surface area of capillary-facing end-feet, constitutes a low-resistance pathway for water movement between these compartments. AQP4 localized to astroglial end-feet around the microvasculature also has a role in draining the interstitial fluid (water and accompanying solute) efflux into the paravenous compartment [13,14,15].

This slow flow of CSF into the brain parenchyma induces a convective flow of interstitial fluid toward the perivenous spaces surrounding the large-caliber draining veins [13]. The drainage of the CSF from periarachnoid spaces and intraparenchymal perivenular spaces (together with interstitial fluid) will follow two pathways: the lymphatic outflow and the venous outflow pathways. The interstitial cerebral fluid forms the glymphatic network, and finally drains in the sinus-associated lymphatics, but a fraction of the subarachnoid CSF also arrives in the dural sinus lymphatics [13,16].

The lymphatic system of the brain was described as a dural network extending from the dural sinuses to both eyes, the cribriform plate via the olfactory bulbs and following the dural arteries and veins into the dura mater, penetrating the skull base with the adjacent vessels through the anatomical foramina, and the transported CSF is discharged into the sheaths of the cranial nerves, finally joining the deep cervical lymph nodes [11]. Therefore, the cranial nerve sheaths represent a common CSF and interstitial fluid outflow pathway from the glymphatic system through the lymphatic dural vessels and from the subarachnoid space (through the dural lymphatic vessels and direct anatomical communications between the cranial nerve sheaths and the subarachnoid space) [13,14,15,16].

Traditionally, it was considered that the arachnoid villi (invaginations of the arachnoid across the dura mater into the lumen of the venous sinus) represent the main pathway of resorption of the CSF from the subarachnoid spaces. Some authors [13] consider that functionally, these arachnoid granulations could be described as “vascular”, centered by a small cortical vein entering the venous sinus, and they are preferentially located in the wall of the transverse sinus, in the area where the Labbé vein joins the sinus, and the rest are the “nonvascular” arachnoid granulations. It was hypothesized that these “vascular” arachnoid granulations represent continuation of the perivenous space of the large-caliber draining veins and receive a part of the CSF and cerebral interstitial fluid from the glymphatic system into the venous blood of the dural sinuses, with another part draining into the dural lymphatics and from there into the general lymphatic circulation [13,16]. The CSF from subarachnoid spaces is drained directly through “nonvascular” arachnoid granulation in large venous sinuses, and from there in the jugular vein and general venous circulation. The proportion of the CSF and interstitial fluid draining through these pathways is not constant, and if one subsystem is not functioning properly (the lymphatic chain or the venous system), the other one will have an increased flow to compensate. From these observations, a combined model of CSF drainage was derived, in which cervical lymphatic exit is the primary site of drainage with the recruitment of arachnoid venous projections under excessive CSF pressure gradients [11,17].

There are medical hypotheses linking the CSF and interstitial fluid with the glymphatic, lymphatic, and venous pathways of drainage and idiopathic intracranial hypertension. First, it was observed (with 3D volumetric MRI measurements) that IIH patients have an excess of cerebral interstitial fluid and CSF in subarachnoid spaces, suggesting a congestion of the glymphatic system [13]. A second observation derives from imaging in cases with IIH, with an excess of CSF observed along the sheaths of the cranial nerves, more frequently described as being the optic nerve sheath dilatation, scleral flattening, and optic nerve tortuosity, that can be explained by the overflow of the lymphatic CSF outflow pathway [18]. The third possibility is related to a stenosis of the transverse sinus, especially at the junction with the Labbé vein, with a backward increase of central venous pressure and inefficient glymphatic venous drainage leading to an excess of cerebral interstitial fluid and overload of the lymphatic CSF outflow pathway [13].

In more than half of the population, the cerebral venous drainage is asymmetric, with predominance of the right side in most cases. An obstruction of the dominant transverse sinus will have a more deleterious effect than of the non-dominant one.

There is a debate as to whether a collapsed wall of the intracranial venous sinus is the cause of intracranial hypertension, or the result of it, but at some point, a cycle of venous hypertension, cerebral swelling, further venous compression, and therefore augmented intracranial hypertension occurs, and this could be a mechanism of worsening. There is a close connection between intracranial pressure and cortical venous pressure, with cortical venous pressure being ~2–5 mmHg higher than intracranial pressure, so there will be a venous flow as long as the ICP does not exceed the inflow venous pressure [19].

The lack of valves in the cranio-vertebral venous system leads the vena cava pressure to be reflected in the CSF pressure, revealing the fact that the venous side of cerebral circulation has much more impact for ICP than the arterial resistance side, but also explaining why an obstacle in venous outflow could be located not only in the intracranial region but also in cervical, thoracal, or abdominal regions.

In case of intracranial obstruction of venous outflow generating an increase of intracranial pressure, the cause of the obstacle could be a focal external venous compression (depressed skull fracture compressing a large venous sinus, periosteal hematoma, tumors), an internal obstruction (most often a sagittal or transverse sinus thrombosis), or a local venous sinus stenosis with idiopathic anatomical local changes; however, sometimes the sinus stenosis could be functional, due to a large volume of arterial blood flowing into the sinus, such as in arteriovenous malformations (AVM) and dural arteriovenous fistulas (DAF) [12]. In these situations of AVM and DAF, the increased venous flow initially produces a maximum distension of the draining vein, but after it reaches the limit of distension, an increased intradural venous pressure will follow (with possible local venous wall changes such as thickening, but also possible thinning and distention producing a local aneurysm), as well as impairment of the mechanisms of CSF absorption with a possible further increase of intracranial pressure until a new level of equilibrium is attained with or without a hydrocephalus development [12,20].

Except for the direct connection between dural venous sinuses of the posterior cranial fossa and sigmoid–jugular venous system, the lateral, posterior, and anterior condylar veins and the mastoid and occipital emissary veins were found to represent another venous connection between the posterior cranial fossa venous sinuses and the vertebral venous systems. All these structures were shown by MR venography. In upright positions, venous drainage preferentially occurs via the spinal venous system, while it is the anterior jugular system in the lying position [21].

An obstruction or difficulty of the venous outflow at the cervical level is possible in particular positions of the neck, such as persistent flexion with lateral rotation, in tight cervical collars, local tumors, and very rarely in jugular compression due to a long styloid process, especially when the opposite venous sigmoid sinus is hypoplastic [22].

Intrathoracic and abdominal causes of venous mechanisms of increased intracranial pressure were associated with positive pressure ventilation in the treatment of chest infection and adult respiratory distress syndrome (ARDS), that can severely raise intrathoracic pressure, severe obesity, and abdominal compartment syndrome with raised intra-abdominal pressure [12].

Severe obesity is indeed frequently encountered in IIH patients, and the hypothesis that it produces a retrograde increase of venous pressure, and further intracranial transmitted, only partly explained the IIH since most of the obese persons with IIH were women, but men with an even higher body mass index (BMI) did not have intracranial hypertension. In some trials, a decrease in BMI was clearly associated with a reduction of headache, papilledema, and CSF opening pressure. The mechanism by which weight loss improves idiopathic intracranial hypertension is not known. The effect of weight in terms of mass alone is not a complete explanation, because BMI and lumbar puncture opening pressure have a non-significant correlation [23]. Obesity is increasingly perceived as an inflammatory disorder, and it is supposed that some cytokines (IL-1β, IL-8, and TNF-α) seem to be associated with IIH pathogenesis [24]. The role of adipokines is not consistently related to IIH in all studies.

## 3. Clinical Manifestations and Diagnosis Criteria of Idiopathic Intracranial Hypertension

The vast majority of patients with IIH are obese (BMI > 30 kg/m^2^) women of fertile age, developing some classical signs of intracranial hypertension dominated by headache and visual disturbances (including papilledema).

The most frequent manifestations associated with idiopathic intracranial hypertension are headache 75–94%, nausea with or without vomiting 72–75%, photophobia, phonophobia, or both 42–73%, transient visual obscurations 68–72%, and pulsatile tinnitus in 52–60% of cases [25].

### 3.1. Headache

Idiopathic intracranial hypertension-associated headaches are of significance to severe intensity, holocranial, frontal, or retro-orbital headaches, but one quarter have moderate and persistent headaches [25,26].

Almost half of the patients have migraine-like headaches, with associated throbbing quality, nausea, photophobia, and phonophobia. There are sometimes some helpful features to raise questions about the diagnosis of “migraine”, such as back-, neck-, or radicular-associated pain, increased severity with Valsalva-type maneuvers, or being worse in the morning and when lying flat [26].

In the Idiopathic Intracranial Hypertension Treatment Trial [27], for the assessment of clinical profiles at baseline for the 165 included patients, the average (SD) headache severity on a scale of 0 to 10 was 6.3, with 5.4% of patients reporting a severity of 10. In 51% of those reporting headache, the headache was either constant or daily (median number of days per month with headache was 12), and 41% reported a premorbid history of migraine (17% had migraine with aura).

There are patients with chronic daily headache-like manifestation, frequently associated with analgesic abuse, but almost two-thirds of IIH patients complain of persisting chronic headache despite a normalization of ICP [28].

A potential risk for missing or delaying the diagnosis are the patients without papilledema, when a long-time refractory headache and resultant neurological disability is in fact due to idiopathic intracranial hypertension. One study found, in evaluating patients with IIH, that about 6% of patients with chronically high ICP have no papilledema [29]. Local anatomical anomalies within the optic nerve sheath might prevent the development of papilledema in IIH patients, since sometimes papilledema could be unilateral, or the moment of the ophthalmology evaluation was before the development of the papilledema. The threshold of CSF pressure required to develop papilledema may depend on individual patient characteristics, and it is possible that those without papilledema have a higher threshold than others, but also the moment of measurement of the CSF opening pressure could find different values of ICP at different moments in the same patient. The opposite situation is encountered when some patients can have higher CSF opening pressures than some with papilledema, but normal cerebral MRI findings, and may simply have chronic daily headaches with coincident elevated intracranial pressure (ICP) at that moment [29]. Due to this particular situation, there are specific diagnosis criteria for pseudotumor cerebri syndrome without papilledema, recommended by Friedman et al. [30] When the usual clinical signs and symptoms of intracranial hypertension are present, the neurological examination is without pathological signs (except for possible 6th nerve palsy), but ophthalmoscopy reveals papilledema, the following steps for a diagnosis of pseudotumor cerebri syndrome are MRI neuroimaging (to rule out intracranial expansive mass, hydrocephalus, cerebral veins or sinus thrombosis, or pathologic meningeal enhancement after gadolinium), a normal CSF composition, but elevated CSF opening pressure at lumbar puncture (≥250 mm CSF in adults and ≥280 mm CSF in children). In the situation of a patient fulfilling the above criteria but without papilledema, he has to present instead of a unilateral or bilateral abducens nerve palsy. If abducens nerve palsy is not present, MRI supportive arguments are required for a probable diagnosis (at least three from empty sella, posterior optic globe flattening, distension of the optical nerve sheath, eventually with tortuosity of the optic nerve, or transverse sinus stenosis).

### 3.2. Visual Features of IIH

Anamnesis should assess the eventual episodes of transient visual obscurations and diplopia and ophthalmology examination should test the visual acuity (each eye separately for the best-corrected distance visual acuity with glasses), color vision with Ishihara’s plates, pupil examination, and visual fields (either a Humphrey’s or Goldmann’s automated perimetry, as confrontational visual fields detect only major defects). The visual field evaluation is considered more sensitive than the decrease of visual acuity in patients with IIH, so it should be carefully assessed at each visit. Dilated fundus examination for optic nerve head and retina is the first very important step to rule out or to confirm a papilledema, which if present should be graded by severity and exclude other ocular causes for disc swelling.

*Transient visual obscurations (TVOs*) refer to sudden loss or shadowy, fogginess, black, white, or grey vision in one or both eyes, lasting for a minute or less than 30 s, sometimes related to a change of position of the body. Increased pressure in liquid from surrounding optic nerve sheaths could compress the thin vascular small arteries and veins at this level, producing a transient ischemia of the optic nerve head [31]. While not specific for raised ICP, the daily occurrence of these symptoms without other explanation (e.g., ischemic amaurosis fugax) is much more frequently encountered in IIH patients.

*Diplopia* in patients with IIH is due in most cases to a sixth nerve palsy, with this nerve being particularly vulnerable to increased intracranial pressure. Diplopia is horizontal, binocular, and if associated with significant abducens nerve palsy, there is a limited abduction of the eye and strabismus. Third and fourth cranial nerve palsies have been described in patients with IIH but are much less frequently encountered.

*Papilledema* is encountered in a vast majority (~95%) of patients with IIH, and because of that it is one of the most important elements of the diagnosis criteria and should be assessed in all patients suspected of having this condition. Sometimes, papilledema may be asymmetrical, with 7% of patients having a difference of 2 Frisen grades or more between the two eyes [27]. In chronic untreated cases, optic disc edema can be followed by optic atrophy due to damage to the retinal ganglion cells. Optic nerve pallor suggests that permanent injury to the optic nerve has occurred. The presence of spontaneous venous pulsations has been considered to exclude the possibility of raised ICP, however the evidence from some studies [32] proved that there are patients with lumbar puncture pressure > 30 cm H_2_O presenting spontaneous venous pulsation, so this situation does not rule out raised intracranial pressure [31].

Sometimes, distinguishing pseudo-papilledema from papilledema requires clinical experience. Pseudo-papilledema may be due to congenitally anomalous discs, optic nerve head drusen, titled myopic discs, inflammation associated with a juxtapapillary optical neuritis, or malignant arterial hypertension. Ophthalmic ultrasound scanning is a useful method to more precisely detect the drusen of the optic nerve head. Optic nerve head drusen was responsible for 6% of misdiagnoses of IIH. B-scan ultrasound is considered diagnostic if there is an area of hyperreflectivity present at the nerve head or if acoustic shadowing of posterior structures of the optic nerve head is detected [33].

Optical coherence tomography (OCT) generates non-invasive, high-resolution cross-sectional images of the retina using a near-infrared light source through undilated pupils. OCT is a useful tool to identify and quantify papilledema, along with some details such as an increase of peripapillary retinal nerve fiber layer thickness and peripapillary choroidal and/or retinal folds, proving a true papilledema [34]. The optic nerve contour, longitudinal assessment of optic nerve swelling, the shape of the back of the eye, and the shape of the scleral opening around the optic nerve are more precisely measurable using OCT. Quantification of the thickness of the retinal ganglion cell layers in the macula is important to alert the clinician that a decrease can reflect irreversible injury to the optic nerve and that the patient is at risk of permanent vision loss [26].

*Visual loss in IIH* could associate a loss in the visual field with a loss of visual acuity. An enlarged blind spot is well-recognized as a common early visual field defect in raised ICP. In the baseline patient group from the Idiopathic Intracranial Hypertension Treatment Clinical Trial, the co-existence of visual field defects with a loss of visual acuity manifesting as an enlarged blind spot and inferior partial arcuate defects were described as the most frequent pathological changes, even in patients with mild disease [27]. Loss of visual acuity is generally accepted to be a feature of advanced disease, but clinicians should be aware that in some cases, we can witness a rapid development of a severe visual loss within the first four weeks from symptoms’ onset in severe cases of intracranial hypertension, prompting surgical intervention to prevent permanent visual deficits [31].

*Pulsatile tinnitus* [27,31] is another common feature of idiopathic intracranial hypertension (in more than half of patients), with two-thirds presenting bilaterally. It consists of hearing a whooshing, whistling, humming, or marching noise, continuously or synchronously with the heartbeats (pulsatile is much more frequent) and is thought to represent an auditory perception of turbulent pulsatile flow in intracranial vessels, or in case of intracranial hypertension, probably secondary to a turbulent flow in transverse venous sinus stenosis [26,31]. It is not specific for intracranial hypertension and also occurs due to other underlying vascular abnormalities (including arteriovenous malformations, arterial stenoses), eustachian tube dysfunction, and other benign causes, but in most cases, remains idiopathic.

## 4. Neuroimaging, Ultrasound, and Lumbar Puncture in Intracranial Hypertension

### 4.1. Neuroimaging

The primary role of brain imaging in idiopathic intracranial hypertension (IIH) is to exclude other pathologies causing intracranial hypertension, but also to sustain the diagnosis with evidence of subtle radiologic findings suggestive of IIH.

**Figure 1:**
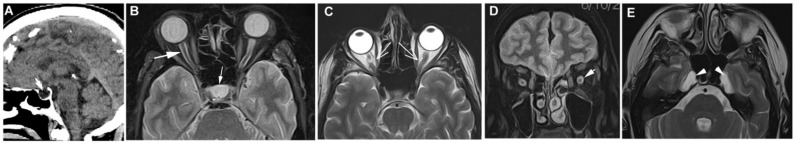
MRI changes in patients with IIH. (**A**) Partial empty sella, T1 weighted MRI. (**B**) Distention of the optic nerve sheath with enlarged cerebrospinal fluid (CSF) spaces surrounding the optic nerve in T2 weighted MRI with fat suppressed sequence (arrow), empty sella also visible (hyperintense signal). (**C**) Noncontrast axial T2 scan reveals that the right optic nerve cannot be entirely displayed along a single plane because the signal of orbital fat obscures the mid-portion of the nerve (“smear sign”). (**D**) Coronal T2 weighted MRI—distention of the optic nerve sheath with enlarged hyperintense CSF ring. (**E**) Axial T2 scan reveals meningoceles involving both of Meckel caves (arrowheads).

Some visible modifications derive from bony erosion from IIH, such as empty sella, meningocele, and foramen ovale widening, while others are due to mechanical deformation from IIH (posterior ocular globe flattening, vertical tortuosity of the optic nerve, transverse sinus venous stenosis), or a limitation of normal flow of fluids in the optic nerve sheath with optic nerve head protrusion and distention of the optic nerve sheath [35,36] (Figure 1).

In a meta-analysis [35] of MRI modification associated with intracranial hypertension, the empty sella had a pooled sensitivity of 62.2% and a pooled specificity of 90.7%, with absolute pituitary area <151 mm^2^ being the most sensitive modification (95.5%). Posterior globe flattening had a sensitivity of 56.3% and specificity of 95.3%, optic nerve head protrusion had a sensitivity of 29.1% and specificity of 97.0%, and optic nerve sheath distension had a pooled sensitivity and specificity of 68.6% and 86.1%, respectively, with individual sensitivity of 78.8% and specificity of 94.2% for a maximum optic nerve sheath diameter > 5.60 mm.

#### 4.1.1. Empty Sella

Empty sella is the most commonly reported imaging finding in patients with IIH, with a sensibility up to 80%, but it is also encountered in the general population. When using the definition based on the cross-sectional area of the sella, the pooled specificity is estimated as 83% (95% CI: 76–90) [18]. When the pituitary gland is not visible on T1 mid-sagittal MRI, being replaced by CSF, without any other lesions, we can assign the case as primary empty sella, while in secondary empty sella, the size of the pituitary gland is decreased compared to the size of sella due to other pathologies, such as pituitary tumor, radiotherapy, drug therapy, head trauma, surgery, or rarely, Sheehan syndrome. Primary empty sella associated with increased intracranial pressure is believed to be related to an intrasellar herniation of arachnoid mater and CSF, which flattens the pituitary gland and remodels the sella turcica, a very slow process which take years.

#### 4.1.2. Changes in the Optic Nerve: Protrusion, Tortuosity, and Sheath Distension

*Optic nerve protrusion* is defined by a focal hyperintensity at the optic nerve head, protruding in the eye, visible on MRI with contrast, and is a representation of papilledema due to increased CSF pressure in the optic nerve sheath and correlates with optic nerve edema seen on OCT as well as the papilledema grade. The detection of contrast enhancement has a smaller sensitivity due to the small dimension of the nerve head and could also be produced in some inflammatory lesions of the optic nerve [36]. In a comparison of neuroimaging findings in patients with idiopathic intracranial hypertension and others with cerebral venous thrombosis, optic nerve head protrusion was present only in patients with IIH, and none in the cerebral venous thrombosis group [37].

*Posterior ocular globe flattening* can be seen on axial MRI at the bulbar insertion of the optic nerve, and is probably produced by increased pressure in posterior juxtabulbar perioptic CSF, compared with the pressure inside the ocular globe. However, this pressure gradient could also be encountered in situations with decreased intraocular pressure and normal ICP, such as ocular hypotony, with similar posterior globe flattening on MRI appearance, an aspect that explains why this sign is not of absolute specificity in IIH. The sensitivity of MRI-detected posterior globe flattening in IIH ranges from 43% to 85% but is still important to be recognized because in some cases, it may precede the installation of papilledema [18].

*Distension of the optical nerve sheaths* is possibly correlated with high CSF pressure in the optic nerve sheath and can be seen on MRI imaging (best in coronal T2 sequences) as a widened ring of CSF surrounding an optic nerve. Definitions for distention of the optical nerve sheaths vary, but a diameter of the CSF ring of more than 2 mm is commonly used [36].

*Tortuosity of the optic nerve* occurs due the fixation of the nerve at proximal and distal points and increased CSF pressure in the optic nerve sheath enlarging the nerve and forcing it to be “kinked”. Detection of tortuosity depends on the MRI slice thickness and orientation, with horizontal tortuosity being less common but more specific for increased ICP than vertical tortuosity [38].

In a dedicated comparison of before and after treatment of patients with IIH with papilledema, in the group with resolution of papilledema, all patients showed improvement in two or more of the MRI characteristics of IIH (height of the midsagittal pituitary gland and optic nerve sheath thickness). Sella configuration, ocular globe configuration, and horizontal orbital optic nerve tortuosity were different between the IIH pretreatment group and controls, but not between controls and the IIH post-treatment group [39].

#### 4.1.3. Slit-like Lateral Ventricles

Narrowing or collapse of the walls of the lateral ventricles, referred to as slit-like ventricles, are very rarely described in association with IIH compared to other causes of intracranial hypertension with associated cerebral edema.

#### 4.1.4. Changes in the Cerebral Venous Sinuses: Transverse Venous Sinus Stenosis

Multiple studies have found that severe bilateral transverse sinus stenosis is present on magnetic resonance venography (MRV) in almost 100% of people with IIH, depending on the definition of transverse sinus stenosis, but as was detailed in pathogenesis, whether transverse sinus stenosis is a primary cause or a consequence of IIH is still under debate. A pathological cycle of stenosis of the sinus contributing to intracranial hypertension with further collapsing of the sinus is possible. This could explain both the reversibility of stenosis with treatment of the increased ICP and the success of the stenting procedure of transverse sinus stenosis in people with IIH. Bilateral transverse sinus stenosis greater than 50% has been found to be a very sensitive imaging marker of IIH [18].

Transverse sinus diameter has been demonstrated to correlate with invasive measured venous pressure gradients in patients with IIH. The degree of stenosis of 30–35%, predictive of a clinically significant pressure gradient in the venous sinuses, was considerably lower than the arterial stenosis at which pathologic hemodynamic alterations occur with a significant pressure gradient. For every 10% increase in the degree of venous stenosis, an approximate increase in the pressure gradient of 3.5 mmHg was seen [40].

In another published study [41], a significant stenosis of both transverse sinuses was found before lumbar puncture in IIH patients, with an average diameter of 1.77 mm of the right transverse sinus and 1.57 mm of the left transverse sinus. After the lumbar puncture, there was a significant increase in all venous sinus diameters, but no correlation between the changes in diameter of the venous sinuses after lumbar puncture and measured CSF opening pressure, or the body mass index. This is to be considered when measurements of sinus diameter are planned a short time after a lumbar puncture.

Accurate assessment of transverse sinus diameter is important to diagnose IIH, but also for treatment decisions and follow-up. MR venography (MRV) can be performed by gadolinium-enhanced or by non-enhanced (including time-of-flight and phase-contrast) techniques. Non-enhanced MRV may be susceptible to effects related to slow or turbulent flow with a risk of underestimating the degree of stenosis, while Gadolinium-enhanced MRV may overestimate the lumen of the transverse sinus, because the dural lining also enhances. In a prospective evaluation, the dural venous sinus diameters were measured in an IIH patient population on two-dimensional time-of-flight MRV and three-dimensional contrast-enhanced (3D-CE), and thereafter MRV were compared with real-time endoluminal measurements with intravascular ultrasound (IVUS) as the reference. The CE-MRV significantly overestimated the cerebral venous sinuses compared to TOF-MRV, while the TOF-MRV sinus measurements were in good agreement with the IVUS measurements [42].

Finally, it is better to use MRV (or CT venography) as a screening tool in case of indication of endoluminal stenting, and if the degree of venous stenosis is significant, the catheter venography will be used for further analysis and measurement of the pressure inside the transverse sinus, before and after the stenosis area, so one can make sure of whether there is a significant pressure gradient or not.

In some, but not all, of these patients, the diameter of optic nerve sheaths decreased after lumbar puncture, and maybe this could be a useful sign for selection of patients for methods of treatment such as CSF diversion, while a close follow-up of visual acuity and visual field for the non-responders and selection of other methods of treatment could be helpful. Mean values of optic nerve diameters were around 2.4–2.7 mm, while mean optic disc elevation in IIH patients was 1.2 ± 0.3 mm in both eyes, and neither were influenced by lumbar puncture CSF removal [42].

In a retrospective study, MRI findings of patients with migraine and IIH (including patients with migraine-like headache) were significantly different, where decreased pituitary gland height, optic nerve sheath distention, and flattened posterior globe were found to be statistically significant (*p* < 0.001) in IIH patients. Bilateral transverse sinus stenosis was also more common in IIH patients than in the control group and the migraine group (*p* = 0.02) [43].

Overall, in a meta-analysis, the bilateral > 50% transverse sinus stenosis with MRI diagnosis had a sensibility of 93.0% and a specificity of 96.4% [35].

### 4.2. The Ultrasound Assessment of Orbital Region

The ultrasound assessment of the orbital region could provide important information for IIH patients in an accessible manner, especially about the mean optic disk elevation as a result of papilledema, the diameter of the optic nerve, and the optic nerve sheaths measured 3 mm behind the papilla. In a published study [44], the mean sheath diameter was 5.4 ± 0.5 mm bilaterally in controls, but with significantly higher values among individuals with IIH, with a mean diameter of 6.4 ± 0.6 mm bilaterally. A cut-off value of a 5.8 mm mean optic nerve sheath diameter for patients with raised intracranial pressure was proposed.

### 4.3. Lumbar Puncture (LP) to Confirm Intracranial Hypertension

LP is mandatory for the diagnosis of IIH but checking for a normal CSF composition should also exclude meningitis or meningeal carcinomatosis. Reference values of ICP and lumbar pressure of CSF were evaluated in a recent meta-analysis [45] which included 9 studies for ICP and 27 studies for lumbar CSF pressure. The measured values for intracranial pressure were −5.9 to 8.3 mmHg in the upright position and 0.9 to 16.3 mmHg in the supine position, while lumbar CSF pressure values were dependent on position, with 7.2 to 16.8 and 5.7 to 15.5 mmHg in the lateral recumbent position and supine position, respectively.

All patients with papilledema and MRI examination without an expanding intracerebral lesion should have a lumbar puncture to measure the CSF pressure [46].

The diagnostic criteria of pseudotumor cerebri [30] require that opening pressure (which should be measured in the lateral decubitus position with stretched legs and without sedative medications, with a manometer zero positioned level with the foramen magnum regardless of patient positioning), should be above 25 cm H_2_O in adults and 28 cm H_2_O in children. However, it is important to note that these cut-offs are not of absolute value, with 2.5% of normal adults having ICP above 25 cm H_2_O in some populational studies and 10% of patients with acute pseudotumor cerebri having ICP less than 25 cm H_2_O [23,47].

Since increased values of ICP may occur intermittently, especially in IIH patients without papilledema, in the presence of suspected intracranial hypertension syndrome with classical clinical presentation and suggestive MRI findings for intracranial hypertension, a second lumbar puncture should be performed if the first lumbar puncture revealed an opening pressure of CSF within the normal range [28].

## 5. Management of IIH

The main principles of management of IIH are targeting the underlying disease if diagnosed, the protection of vision, and to reduce the headache morbidity. During the last decades, a combination of lifestyle measures, drug treatments, and in selected cases different surgical and endovascular interventional therapies were developed. Before deciding the treatment strategy of an IIH patient, the first step is to check if other known factors associated with increased intracranial hypertension are present, and if identified, they should be addressed. According to Friedman [30], the most frequent causes of pseudotumor cerebri are cerebral venous abnormalities (cerebral venous sinus thrombosis, arteriovenous fistulas, venous decreased CSF absorption from previous intracranial infection or subarachnoid hemorrhage, bilateral jugular vein thrombosis or surgical ligation, increased right heart pressure, superior vena cava syndrome, and the risk factors for cerebral venous and sinus thrombosis, such as middle ear or mastoid infection, hypercoagulable states). Exposure to specific medication and abuse of some substances is another possible cause (tetracycline, minocycline, doxycycline, nalidixic acid, vitamin A excess and retinoids, isotretinoin, lithium, chlordecone), but also some endocrine dysfunction (related to human growth hormone, thyroxine—in children, Addison disease, hypoparathyroidism, anabolic steroids, but also withdrawal from chronic steroid treatment). Obesity is frequently associated with hypercapnic status due to sleep apnea or Pickwickian syndrome. Finally, other diseases such as anemia, renal failure, and Turner and Down syndrome are sometimes associated with high ICP values.

### 5.1. Weight Reduction

A large majority of patients with pseudotumor cerebri are overweight, but especially obese (90–95%), with 90% of them being women of fertile age. In the year preceding a diagnosis of IIH, many patients had a weight gain of about 5–15%, but in a cohort study, a weight loss of 15% was associated with a significant reduction of ICP (mean 8 cm H_2_O), headache, and papilledema, although a correlation between the amount of obesity and CSF pressure was not always found [46,47].

Considering the association between weight gain and IIH recurrence, a weight management plan should be a long-term, sustainable target. Once definite IIH is diagnosed, all patients with a BMI > 30 kg/m^2^ should be counselled about weight management with a low-energy diet [48] and integrated in community weight management programs. For those without significant results from community management programs, bariatric surgery is another option and has an increasing role. A comparison between these two options is now available, following the results of a recent randomized study [49] in these obese patients. In this randomized clinical trial of 66 women with idiopathic intracranial hypertension and a body mass index of 35 or higher, bariatric surgery was superior to a community weight management intervention in decreasing intracranial pressure at 12 and 24 months (mean difference 6 and 8.2 cm, respectively, in CSF opening pressure between groups), with weight loss being more significant in the bariatric surgery group (mean 21.4 kg at 12 months and 26.6 kg at 24 months).

### 5.2. Drug Treatments

Acetazolamide is considered the drug of choice for IIH patients. The mechanism of acetazolamide’s effect in reducing the CSF production is thought to be related to inhibition of carbonic anhydrase that causes a reduction in the transport of sodium ions across the choroid plexus epithelium. It has been shown to reduce CSF production by 6% to 50% using a relatively high dosage in humans.

Two double-blind, randomized trials with acetazolamide in patients with IIH were analyzed in a 2015 Cochrane review [50]. The first one was a pilot study with 25 patients per arm [51] and the second one was The Idiopathic Intracranial Hypertension Treatment Trial (IIHTT) [52] with 165 participants (86 patients in the acetazolamide group and 79 in the placebo control). Both trials’ participants had a mild form of disease, meaning that evidence for the use of acetazolamide in participants with moderate to severe visual loss is lacking. In the IIHTT, compared with the placebo, the treatment with acetazolamide demonstrated an improvement in visual perimetric mean deviation, papilledema grade, visual related quality of life, and CSF opening pressure at 6 months, but it did not find a benefit in visual acuity or symptomatic headache relief. The IIHTT used a maximal dose of 4 g daily (but only 44% of participants achieved 4 g/day, with the majority tolerating 1 g/day). The usual starting dose of acetazolamide is 250–500 mg twice a day, and a slow dose increase thereafter within the limits of tolerance. The most frequent adverse effects of acetazolamide include increased risk of diarrhea, dysgeusia, fatigue, nausea, paresthesia, tinnitus, vomiting, depression, and rarely, renal stones. Overall, the Cochrane review considered the two studies too small and not representative for the whole population of patients with IIH, and they did not allow quantification of either relative or absolute benefit of treatment, so there is still a need for a large RCT that can provide this information.

A possible alternative to acetazolamide is topiramate, which theoretically could have some advantages: it is also a weak carbonic anhydrase inhibitor, has some migraine preventive effects (and some IIH patients have a migraine-type headache or a previous migraine), and induces appetite suppression, leading to potential weight loss.

An open-label study of 40 patients with idiopathic intracranial hypertension compared topiramate (100–150 mg per day) and acetazolamide (1000–1500 mg per day) [53]. The main outcome was the visual field-graded deficit, which at the 3-month, 6-month, and 12-month visits, improved without a significant difference in the 2 groups. The topiramate group had the advantage of an associated significant weight loss. A prominent relief in headache was reported after a mean treatment period of 3.75 months in the topiramate and 3.3 months in the acetazolamide group, while papilledema grades began to regress after the second month with a mean treatment period of 5.5 months for the topiramate and 5.1 months for the acetazolamide group, but the difference did not reach a significance level. Patients need to be cautioned about potential side effects of topiramate, such as depression, cognitive slowing, reduction of the efficacy of oral contraceptives, and some potential for teratogenic effects.

For patient with previous migraine overlapping with an increased intracranial pressure syndrome, some classical drugs used for migraine attack treatment are still useful (triptan acute therapy used in combination with NSAID or paracetamol and antiemetics), avoiding long-term abuse of analgesics. Topiramate could be used for the prevention of migraine attacks, but in some cases for patients with associated depression, venlafaxine could also play a positive role, avoiding the risk of weight gain seen with betablockers, tricyclic antidepressants, or sodium valproate.

### 5.3. Surgical and Interventional Therapeutic Methods

#### 5.3.1. CSF Diversion Methods

CSF divergence can be the logical step to reduce the disturbed balance between secretion and drainage of CSF, with an increase in total intracranial fluid content, to compensate for other possible links such as intracerebral venous and glymphatic system compression secondary to intracranial hypertension, but also large venous sinus compression with extrinsic stenosis. This secondary venous sinus stenosis can lead to a further increase in ICP, which creates a vicious cycle where the increase in ICP produces a worsened secondary sinus stenosis and higher venous pressure [51,54]. There is evidence that drainage of CSF with a reduction of ICP can also reverse the transverse sinus collapse in patients with idiopathic intracranial hypertension [54,55].

In patients with a high risk for visual loss, CSF divergence can be sight-saving. If the surgical procedure is likely to be delayed for 24–48 h, it is possible to insert a lumbar drain at the time of the lumbar puncture. Other methods are a lumbo-peritoneal shunt, where a catheter is inserted into the subarachnoid space at the lumbar spine and the distal part enters the peritoneum, but this method is not appropriate in the case of patients with low-lying cerebellar tonsils, as there is an increased risk of tonsillar herniation following the shunt. A ventriculoperitoneal shunt diverts CSF from the lateral ventricle to the peritoneum, and a ventriculoatrial shunt diverts CSF from the lateral ventricle to the atrium of the heart, while a ventriculopleural shunt diverts it to the pleural cavity, but the last two techniques are less used. The surgical risks include shunt malfunction, infection, and over-drainage. Due to the lower reported rate of shunt revisions per patient, the ventriculoperitoneal route should be the preferred CSF diversion procedure for visual deterioration in IIH [46].

The results of CSF drainage methods were evaluated through some retrospective case series and a meta-analysis of them. In a retrospective study [56] with 53 cases of CSF diversion (in most cases, lumbo-peritoneal shunt), significantly fewer patients experienced declining vision and a visual acuity improvement at 6 and 12 months, although headache continued at 6, 12, and 24 months (68%, 77%, and 79%, respectively). Additionally, post-operative low-pressure headache occurred in 28%. Shunt revision occurred in 51% of patients, with 30% requiring multiple revisions.

In a meta-analysis of 17 studies with a CSF diversion including 435 patients [57], the headache improved in 80% of patients, papilledema in 70%, and visual acuity in 54%, but 43% needed another additional surgical procedure, and most of them were related to revision of the shunt for shunt obstruction or failure, malposition, valve dysfunction, or low-pressure headache. The other complications were subdural hematoma, tonsillar herniation, radicular pain, and CSF fistula. The rate of major complications was 7.6% (shunt infection, tonsillar herniation, subdural hematoma, and CSF fistula) [57].

Overall, different methods of CSF shunting have benefits in reducing CSF pressure, in improving visual deficits, and in preventing visual loss, but this is not the procedure of choice for intracranial hypertension headache prevention or treatment.

#### 5.3.2. Venous Sinus Stenting

Stenosis, as previously defined by Marmarou et al. [58], is an acute reduction in the caliber of the venous vessel by at least 40%, while a hypoplastic sinus is decreased in diameter on average by 40% compared to the dominant venous sinus. There is an increased prevalence of right transverse sinus (RTS) dominance, being larger on average than left transverse sinus (LTS).

The DSA estimated pattern of venous drainage in a cohort of patients showed that a right-side dominance is the most prevalent pattern of drainage (49%), while codominance occurs in 43% and left-side dominance in 8%. A complete unilateral drainage is seen in 19% (right) and 1% (left) of patients [59].

Dural sinus stenosis is generally described as intrinsic or extrinsic, although some patients may have both. An intrinsic obstruction pattern is a focal filling defect secondary to arachnoid granulation (often round/oval touching a dural sinus wall), whereas an extrinsic stenosis may be related to local hypertrophic scarring or related to elevated intracranial hypertension, which itself compresses the wall of the sinus (in this situation, the stenosis appears often as a long, tapered, and smooth narrowing) [60].

The arachnoid granulations were present in the superior sagittal sinus in 50% of patients, with a 7% incidence of sinus stenosis. Most of the stenosis in the sinuses was due to the arachnoid granulations (77% on LTS and 71% on RTS) [60]. Overall, only 6% had decreased flow through both sinuses, either by bilateral transverse sinus stenosis or unilateral stenosis with contralateral hypoplastic sinus. Contrary to non-IIH patients, bilateral transverse sinus stenosis is prevalent in 90% of IIH patients [60,61].

The hypothesis linking the dural venous sinus stenosis and IIH leads to the conclusion that solving the stenosis using angioplasty and placement of a stent could serve as another method for treatment of the disease, especially in patients who are unresponsive to medical therapy. Research evaluating this therapy began with Higgins and colleagues, in 2003, who demonstrated a clinical improvement in 7 of 12 medically refractory IIH patients treated with venous sinus stenting, 5 of whom experienced complete resolution of symptoms [62].

Recently, a new proposal emerged [63] for changing the name of idiopathic intracranial hypertension to chronic intracranial venous hypertension syndrome (CIVHS) and to change the diagnostic criteria with a new, combined criteria which requires opening pressure on lumbar puncture or intracranial pressure > 25 cm H_2_O and elevated superior sagittal sinus pressures (>18 mmHg) in the absence of an intracranial mass lesion. Based on central venous pressure and the presence of significant venous sinus stenosis, four subtypes of CIVHS were proposed: (1) Central-type patients have elevated central venous pressure without concomitant venous sinus stenosis, whereby morbid obesity or cardiorespiratory disease result in significant central venous pressure elevations with subsequent elevations in intracranial venous pressures. These patients account for about 25% of patients with IIH, are not candidates for venous sinus stents, and tend to respond to weight loss or furosemide to reduce central venous volume and therefore central venous pressure. (2) Craniocervical-type patients demonstrate pathologic venous sinus stenosis with low–moderate CVP, wherein the venous outflow obstruction at the venous stenosis manifests as the primary driver of elevated intracranial venous pressure. These patients are often excellent candidates for venous sinus stents. (3) Mixed-type patients are the largest subset of patients and demonstrate both pathologic venous sinus stenosis as well as moderate to high venous sinus stents, wherein both are independent and additive drivers of high intracranial venous pressure. (4) Post-thrombotic patients demonstrate impaired intracranial venous outflow due to chronic venous sinus thrombosis, and are the rarest [63,64].

Evaluation of venous cerebral circulation should especially detail the location, extension, and degree of stenosis of dural venous sinuses, if possible, with noninvasive imaging such as CT venography, or contrast-enhanced magnetic resonance venography, which can also demonstrate intrinsic/extrinsic stenoses. A direct retrograde catheter venography with venous manometry is required to determine whether a venous pressure gradient exists across the stenotic segment. Pressures are measured from the segments of the superior sagittal sinus, bilateral transverse sinuses, sigmoid sinus, jugular bulb, and cervical internal jugular. A stenosis is considered clinically significant if a trans-stenotic gradient above a certain level is found between the proximal and distal segments of the evaluated venous stenosis. Normal gradients between the superior sagittal sinus and the jugular bulb range between 0 and 3 mmHg in healthy patients. A universally recognized cut-off value for the trans-stenotic gradient to determine which patients are appropriate candidates for stenting has not been established, although most practitioners prefer 8 mmHg for the selection of patients for stenting, and the maximum values are frequently more than 10 mmHg [65].

The likelihood for a cerebral venous pressure gradient is almost 5 times higher (OR 4.97, 95% CI 1.71–14.47) in patients diagnosed with IIH, but very low in patients with a preexisting shunt (OR 0.09, 95% CI 0.02–0.44) and absent in those with normal ICP [66].

There are opinions that the trans-stenotic venous gradient should be measured before and after lumbar puncture, and confirmation of indication for stenting of the venous stenosis remains in patients without significant post-puncture improvement of the gradient. Secondary venous sinus stenosis due to external compression within an intracranial hypertension syndrome may respond to a decrease of ICP after lumbar puncture, while gradient pressure due to intrinsic stenosis is thought to be less responsive to a post-lumbar puncture decrease of ICP [65]. However, the secondary extrinsic venous sinus stenosis can lead to a further increase in ICP, which creates a vicious cycle, a proven fact that, together with case series demonstrating the efficacy of stenting in reducing ICP in patients with extrinsic stenosis, supports the consideration of the procedure in both extrinsic and intrinsic venous sinus stenosis. The cerebrospinal fluid opening pressure and trans-stenotic pressure gradient were significantly decreased post-intervention. The stent reinforces the walls of the transverse sinus and increases its resistance to extramural compression, restoring a more physiologic gradient of venous outflow. This helps with lowering the ICP by preventing the development of upstream venous congestion [64,65].

In a meta-analysis of 32 eligible studies, a total of 186 patients were included for predicting outcome values of pressure gradients [67]. Patients who had favorable outcomes had higher mean pressure gradients (22.8 ± 11.5 mmHg vs. 17.4 ± 8.0 mmHg, *p* = 0.033) and higher changes in pressure gradients after stent placement (19.4 ± 10.0 mmHg vs. 12.0 ± 6.0 mmHg, *p* = 0.006) compared with those with unfavorable outcomes. In a multivariate stepwise logistic regression controlling for multiple factors, the change in pressure gradient with stent placement was found to be an independent predictor of favorable outcome (*p* = 0.028). Using a pressure gradient of 21 mmHg as a cut-off, 94.2% of patients with a gradient > 21 mmHg achieved favorable outcomes, compared with 82.0% of patients with a gradient ≤ 21 mmHg (*p* = 0.022), but the high percentage (82%) of patients with favorable outcome with pressure gradient ≤ 21 mmHg made it difficult to choose this value as a definitive indication for intervention [67].

Ultimately, venous manometry is the most important parameter to select a venous stent indication as a treatment after diagnosis of significant intracranial hypertension with progressive symptoms/vision loss and an obstructive venous outflow pattern (isolated sigmoid sinus stenosis, bilateral transverse/sigmoid sinus stenosis, or ipsilateral transverse/sigmoid sinus stenosis with contralateral transverse/sigmoid sinus hypoplasia or absence of sinus), without a response to weight loss and maximal medical therapy, after careful comparison of other alternative surgical methods and patient particularities.

Prior to stent placement, patients are provided with seven days of aspirin 75 mg and clopidogrel 75 mg daily or loading doses of aspirin 325 mg and clopidogrel 600 mg. Trans-stenotic maximal venous pressures and pressure gradients are reconfirmed prior to placement of the venous stent. For venous sinus stenosis, the stents need to be self-expanding with adequate radial force to overcome any external stenosis from elevated ICP and long constructs to ensure they extend >10 mm pre- and post-stenosis [68].

Post-stenting venograms are performed to look at the drainage patterns. A CT scan is performed to rule out an intracranial hemorrhage. Patients will be on dual-antiplatelet therapy for one month and single-antiplatelet therapy for at least 3–6 months [46,69]. An array of stents are used for this purpose, such as Zilver 518 of 10 mm-diameter (Cook Medical), the SMART 10 mm-diameter stent (Cordis), Protégé Everflex (Covidien), Precise (Cordis), and Wallstent (Boston Scientific), but the list is continuously expanding [68]. An ongoing study (estimated study completion date January 2024), “Operative Procedures vs. Endovascular Neurosurgery for Untreated Pseudotumor Trial OPEN-UP”, which compares venous sinus stenting versus ventriculoperitoneal shunt placement, uses the Zilver stent (ClinicalTrials.gov identifier NCT number NCT02513914).

There are no studies specifically reporting on synchronous bilateral transverse sinus stenting for patients with IIH. The vast majority of published studies performed unilateral stenting of the most stenotic transverse sinus, with good procedural outcomes [69].

The clinical outcomes of dural sinus stenting are related to headache, but especially improvement of visual parameters (visual acuity, papilledema), and as a secondary achievement, the improvement or disappearance of the tinnitus. In the published meta-analysis and retrospective reviews of case series (Table 1), there was a consistent benefit with respect to these parameters: headache subsided or was significantly improved in 73–93% of patients, papilledema in 68–100%, visual acuity and visual field improvement in 70.3–86.5%, and tinnitus in 84.5–100% of patients [57,61,65,70,71,72,73].

In a dedicated meta-analysis of 395 patients [72] with available follow-up data on stenting outcome (mean of 18.9 months), the stent survival and stent-adjacent stenosis rates were 84% (95% confidence interval (CI): 79–87%) and 14% (95% CI 11–18%), respectively. In a retrospective case series [74], overall, 25.9% of patients underwent further surgical intervention following venous sinus stenting, including 6.2% of the total number of patients who repeated a venous sinus stenting procedure and 22.2% who underwent cerebrospinal fluid shunting. Except for restenosis, adjacent stent stenosis or stent thrombosis represent other parts of the treatment failure. The opposite-side venous sinus stenosis is explained by unexpected development of contralateral transverse sinus stenosis after previous stenting of the culprit stenotic sinus. It is assumed that the post-stenting reduction of internal pressure in the opposite transverse sinus—after a large part of venous flow is again directed through the stented dilated sinus—will favor shrinking of the non-stented sinus, and in the most extreme situation, even an occlusion, with re-initiation of a negative vicious cycle of increasing intracranial hypertension and venous collapsing.

The rate of major complication of the procedure (intracranial hemorrhage, subdural hematoma, and subarachnoid hemorrhage, permanent neurological disability) varied from 0% to 1.5–2.9% [57,61,74]. Some minor complications in 3.4–5.4% of cases [57,61,68] were most often related to angiography, or were transient and described as retroperitoneal hemorrhage, retroperitoneal hematoma, femoral artery pseudo-aneurysms, femoral vein thrombosis, neck hematoma, transient hearing losses, transient headache, urinary tract infection, syncope, and post-stenting headache. The mechanism of cerebral venous sinus stenting headache (clinically different than that produced by IIH) remains unknown. Suggestions have included: mechanical stimulation of the venous sinus wall during the procedure, local toxicity, a chemical reaction to the dye, or inflammatory changes, a lower pain threshold, variation in the locations of the hypersensitive pressure receptors of the venous vessel walls, or changes in the pain threshold in the context of physical and psychological stress related to the procedure [75]. According to ICHD-3 criteria [76], cerebral venous sinus stenting headache is defined as any new headache or previous headache that has significantly worsened within one week after jugular or cranial venous stenting has been performed, is ipsilateral to the stenting, or bilateral, and is not better accounted for by another diagnosis. In a dedicated analysis of post-cerebral venous stent headache of 48 patients [75], the headache was present in 29%, of which 92.9% were on the same side as the stent, mild and moderate, in most cases occipital-located, persisted for less than 3 days in 42.8%, for 3 days to 3 months in 28.6%, and for longer than 3 months in 28.6%.

Compared with the other interventional/surgical procedures for IIH, the rate of improvement of the associated tinnitus is higher with stenting of venous sinus stenosis. Pulsatile tinnitus in the majority of patients with IIH is described as a whooshing sound synchronous with their pulse. This has been attributed to turbulent venous flow through a stenotic segment, and compression of the ipsilateral jugular vein may result in its cessation. A 3 T/four-dimensional (4D) flow magnetic resonance imaging with fast-field echo study [77] proved that on the tinnitus side, all patients had sigmoid sinus wall dehiscence, and patients with transverse sinus stenosis showed significantly higher maximum flow velocities than those without transverse sinus stenosis. A jet-like flow in the stenosis and downstream of the stenosis was observed in all patients with transverse sinus stenosis. Since increased prevalence of sigmoid sinus diverticulum/dehiscence and transverse sinus stenosis in idiopathic intracranial hypertension did not correlate with the presence of pulsatile tinnitus in another study [78], there are probably other hemodynamic factors such as intra-stenotic maximum velocity dependent on the local trans-stenotic pressure gradient and complex flow patterns, such as vortex flow, turbulent flow, and helical flow, that are better correlated with pulsatile tinnitus [79].

#### 5.3.3. Optical Nerve Sheath Fenestration (ONSF)

There are very few dedicated studies on ONSF in IIH patients, and in general, they are retrospective case series. The mechanisms through which ONSF reduces the optic nerve consequences of high ICP are not clear. The MRI-proven increase of optic sheath thickness and increased tortuosity of the optical nerve could lead to the idea that the ONSF exerts its action by local fistula formation between the nerve sheath and dura, with slow drainage and resorption of the excess of fluid in the orbital tissue, or a local hypertrophic scarring in the first segment of the optic nerve with a further limitation of transmission of increased pressure in the distal optic nerve and papilla [31].

ONSF was more often considered in patients with asymmetrical papilledema and mainly in patients where headache is not significant, because of the more modest improvement rate compared to the other treatment methods, or in a situation when CSF diversion surgery is considered to carry a high risk or is contraindicated.

The meta-analysis of Satti et al. of optic nerve sheath fenestration included 712 patients. Post-procedure, there was an improvement of vision in 59%, headache in 44%, and papilledema in 80%, and 14.8% of patients required a repeated procedure with major and minor complication rates of 1.5% and 16.4%, respectively [57]. The major complications following ONSF included retinal artery occlusions, retrobulbar hemorrhage, traumatic optic neuropathy, orbital apex syndrome, orbital cellulitis, and manifest strabismus, while minor complications included transient double vision, anisocoria, conjunctival problems/cysts, and optic nerve hemorrhages [46,57].

Unilateral ONSF in a case series of 62 patients [80] significantly decreased the grade of papilledema in both ipsilateral (operated) and contralateral (unoperated) eyes. The reduction of the papilledema and the stability of the visual field in the contralateral (not operated) eye suggest that bilateral ONSF may not always be necessary in patients with bilateral visual loss and papilledema due to IIH, and probably that the cerebrospinal fluid that filters through the dural opening fistula into the orbit produces a subsequent decrease of subarachnoid CSF pressure, and this could explain the therapeutic effect. Unilateral superomedial transconjunctival ONSF was the single treatment method in another small retrospective case series of patients with IIH [81]. Visual acuity, perimetric mean deviation, papilledema grade, and optic nerve head elevation were significantly improved after 6 months in both the operated and non-operated eye. Optic nerve head elevation and visual field testing with automated perimetry could be viable biomarkers for assessing early treatment efficacy after ONSF.

## 6. Conclusions

The accurate diagnosis of idiopathic intracranial hypertension is essential as visual deterioration due to papilledema may be irreversible. An increase in incidence is expected in the future because of the rising levels of obesity. Although the mechanisms of this heterogeneous syndrome are complex and not all of them are fully elucidated, there are advances in the understanding of idiopathic intracranial hypertension and improvements in diagnosis, based on current available criteria, including specific CSF pressure levels. The therapeutic options include reducing weight strategies (low-calorie diet and bariatric surgery), medication (especially acetazolamide and topiramate), and in selected severe cases, some interventional therapies such as ventriculoperitoneal shunts, optical nerve sheath fenestration, and intracranial venous sinus stenosis stenting, with a possibility to adapt them to the patient’s clinical profile for the best results.

## Figures and Tables

**Table 1:** Improvement of clinical outcomes after dural venous sinus stenting.

Authors/Parameter	Headache	Papilledema	Visual Performance	Tinnitus
Satti S.R. et al., 2015 [57]	83%	97%	78%	-
Leishangthem, L et al., 2018 [61]	82%	92%	78%	-
Dinkin M. et al., 2019 [65]	73%	68%	74%	85.1%
Ahmed R.M. et al., 2011 [70]	93%	100%	-	100%
Starke R.M.et al., 2015 [71]	78.3%	94.4%	86.5%	92.9%
Saber H.et al., 2018 [72]	77.6%	85.8%	70.3%	84.5%
Liu X. et al., 2019 [73]	**-**	**-**	**84.2%**	**-**
